# Supplementary material for: Are carbon emissions trading and green financial instruments synergistic? -Comprehensive quantitative research based on content analysis
Source: PLoS One. 2024 Mar 7;19(3):e0298601. doi: 10.1371/journal.pone.0298601 (PMC10919601; doi:10.1371/journal.pone.0298601)
Supplement: S1 Data — (ZIP) [file pone.0298601.s001.zip › Data/地级市Z_F工作报告.zip!/╣π╢1⁄2/╢1⁄2▌╕╩╨/└┤╫╘╣1⁄2╓┌║┼í╛┬φ┐╦╩2╛▌═°í┐_2015.docx]

各位代表：

现在，我代表市人民政府，向大会报告2014年政府工作，对2015年工作提出建议，请予审议。并请政协各位委员和其他列席人员提出意见。

2014年工作回顾

“看似寻常最奇崛，成如容易却艰辛。”一年来，全市上下在上级和市委的正确领导下，以党的十八届三中、四中全会精神为指导，积极应对各种不利因素的影响，聚力改革攻坚、项目攻坚、效能攻坚，扎实做好各项工作，较好地推进了市第十五届人大四次会议确定的年度目标任务。

在经济下行压力加大的一年里，我们以多管齐下的积极举措，保障了经济发展稳中有进。面对外部需求不振、国家政策收紧、结构调整阵痛等因素的叠加，出台有力措施扶持实体经济、稳定外贸增长、促进商贸和房地产等产业健康发展，努力推动在平稳发展基础上加快转型升级。预计全市生产总值5881亿元，同比增长7.8%，快于全国平均水平0.4个百分点。市一般公共预算收入455.2亿元，增长11.2%。人民币存款余额突破9000亿元。规模以上工业企业内销比重首次超过外销。海博会、加博会、国际科技合作周等重要活动成功举办。新型研发机构建设、电机能效提升、节能减排等工作得到上级高度肯定。在社科院发布的蓝皮书中，东莞城市综合经济竞争力排全国第12位。福布斯2014中国城市创新力排行榜上，东莞居第11位。

在改革发展任务艰巨的一年里，我们以先行先试的争先意识，实现了关键领域改革的良好开局。200多项改革陆续铺开，在全国率先形成系统化的商事制度后续监管模式，率先发出跨境电商货物通关第一票，率先推行外商投资“多证联办”，率先启动项目投资建设直接落地改革，社会投资项目审批时间缩短三分之二。东莞获批国家新型城镇化综合试点城市，顺利通过全国质量强市示范城市验收，争取了一批先行先试改革项目，争取了更多的政策红利。重大项目超过七成动工，一批项目建设速度创下新纪录，为城市经济发展注入了强大动能。在北京师范大学发布的2014中国地方政府效率排行榜上，我市在全国54个重点城市中排名第一。

在深层矛盾接连爆发的一年里，我们以及时果敢的应对处置，倒逼了社会治理水平提升。面对长期积累社会问题的集中凸显，我们痛定思痛，沉着应对，以“扫黄”歼灭战为抓手铲除不健康产业生态，以处置“裕元事件”为契机完善社保、公积金缴存机制，以应对“丐帮”失实报道为切入点加强流浪乞讨未成年人救助，全方位改进城市管理、权益保护、公共安全等方面的不足，努力把问题暴露带来的压力，转化成倒逼整改、长效治理的动力，促进了社会治理法治化水平全面提升。

在深入开展群众路线教育实践活动的一年里，我们以对“四风”问题的坚决整治，促进了作风改进和民生改善。八项规定、三严三实等要求全面落实，一批形式主义、官僚主义、享乐主义和奢靡之风问题得到集中整改，切实解决服务群众“最后一公里”问题，密切联系群众日益成为机关新风尚。十件实事圆满完成，扶贫、公交等民生工程扎实推进，城市社区居家养老实现全覆盖，基本养老金连续提升，普通高考总录取率等四项高考指标实现全省“三连冠”，医保满意度居珠三角首位，村组资产负债率降至历史新低，群众在发展中得到更多实惠。

一年来，我们主要抓了以下工作：

——着力扶持以先进制造业为核心的实体经济发展。出台50条措施扶持实体经济。开展千干扶千企活动，积极缓解企业融资难、用工难等问题。先进制造业增加值增长13.9%，占规模以上工业的47%，比2013年提高2.4个百分点。新增规模以上工业企业145家。规模以上工业企业内销比重提高到52%。百亿元企业达10家，五百亿元企业实现零突破。一批战略性新兴产业初具规模，智能手机年出货量2.3亿部，占全球的17.7%。从事云计算应用的企业超2000家。物联网及相关产业年产值680亿元。东莞成为第二个国家级两化深度融合暨智能制造试验区。新增境内外上市企业7家，总数达29家。新增全国股转系统挂牌企业15家、区域性股权交易中心挂牌企业近200家，均居全省地级市第一。实体经济质量效益进一步提升。

——以超常规力度解决“三率”问题，促进重大产业项目建设全面提速。坚持每月会诊督促重大项目建设，落实市领导包干、“五个一”、明白卡、销号制等制度，全力推进重大项目建设。全市新开工重大项目45个、总投资576亿元。截至2013年底的156个重大项目开工率从年初的42.9%提高至71.8%。一批项目建设投产速度创下新纪录，大朗三星项目从签约到投产仅用8个月，松山湖记忆科技总部提前7个月开工。累计24个项目建成投产，一批项目逐渐释放产能。组团赴东南亚、南美、日本、以色列、土耳其等地开拓市场、加强招商。新引进一批总投资558亿元的重大项目，发展后劲不断增强。

——加快重大平台和重要基础设施建设。水乡特色发展经济区管委会获省批准成立，挂影洲中心涌等8个示范片区和基础项目开工，水乡大道改造等工程竣工。粤海银瓶合作创新区上升为省级战略，首个产业项目动工，总投资100亿元的环普工业园项目签约。东莞港区集装箱达288万标箱，增幅连续三年保持全国超百万标箱港口第一位。东莞保税物流中心（B区）进入全国B型保税区四强。松山湖高新区主要经济指标增长25%以上，辐射带动作用不断增强。生态产业园引资106亿元。长安新区完成总体规划编制。松山湖高新区与生态产业园、长安镇与长安新区实行统筹发展。省市重要基础设施项目建设超额完成年度目标，带动全社会固定资产投资1427亿元，增长10%。地铁2号线车站全部封顶，隧道全线贯通。虎门二桥正式动工，东部快速改造完工。东莞篮球中心正式投入使用，市民艺术中心、工人文化宫完工。建成110千伏以上输变电工程15项。4G网络基本实现全覆盖。

——以新型研发机构、“机器换人”等为抓手，增强科技创新能力。大学创新城一期工程基本竣工。全市新型研发机构增至23家，居全省第三。启动“机器换人”行动计划，市财政连续三年、每年安排2亿元对企业进行资助。设立机器人产业基地和创投基金。成立新能源汽车联盟。新引进省创新科研团队7个，总数22个，在全省均排第三位。新增国家高新技术企业80家、企业研发机构657家，总数分别达755家和1593家。发明专利授权量居全省第三。350多家企业开展电机能效提升，年节电量超2.6亿千瓦时。散裂中子源首台设备安装成功。预计研发经费支出增长12%，占GDP比重2.1%，连续六年提升。

——深入推进商事制度、项目投资、外商服务管理等改革。积极探索电商集群注册、住所信息申报、加强协同监管等商改新举措，市场主体达63万户。大力推动项目投资审批体制改革，探索直接落地改革试点，社会投资项目落地时间从18个月压减至7个月。推行外商投资市场准入“多证联办”改革，企业仅用3、4个工作日即可领齐相关证照。深化审批制度改革，行政审批和日常管理事项累计压减幅度分别达68.1%、67.5%。深化政府机构改革，调整理顺物价、商贸、卫生、计生等职能，政府工作部门压缩调整为32个。社会信用体系建设加快推进。医疗、公交、户籍等民生领域改革进一步深化。

——成功举办海博会等大型展会，对外开放取得新成绩。圆满举办省委省政府交办的首届21世纪海上丝绸之路博览会，开辟了广东及东莞与沿线国家交流合作的重要平台。加博会、漫博会、台博会、国际科技合作周等影响力不断扩大。构建开放型经济四大体系，实施加工贸易增效计划，出台稳定外贸增长37条措施。全市进出口总额1625.3亿美元，增长6.2%，增速在全国外贸总额前五名城市中排第一位。承办中国（广东）——马来西亚经贸合作交流会。推动成立中英低碳产业园。与罗马尼亚布里扎市、捷克奥帕瓦市和德国乌波塔尔市结为友好城市。

——扶持电子商务等新兴业态发展。成功创建国家电子商务示范城市。全市电商主体达5.4万户，电商交易额增长26%，阿里巴巴网络收货量和发货量分别居全国第七和第二。与阿里巴巴牵头成立的菜鸟网络签约，建设中国智能骨干网节点项目和华南区域O2O体验式购物中心项目。四成多中小企业应用电子商务。在海关总署支持下，率先启用跨境电商服务平台、建设跨境电商公共监管仓，规划建设一批跨境电商园区，东莞速卖通跨境电商交易额升至全国第七。成立城市共同配送标准联盟，获准设立清溪保税物流中心（B型）。东城万达、长安万科等城市综合体相继开业，商业生态环境进一步完善。社会消费品零售总额1615亿元，增长8.7%。

——加大节能减排与生态建设力度。推进国家节能减排财政政策综合示范城市建设，全面启动三年总投资350亿元的示范项目建设计划。启动水乡“两高一低”企业整治与退出。推进土地生态利用，整合盘活土地近5万亩。实施大气污染防治行动。超额完成黄标车淘汰任务，环保限行范围进一步扩大。投放4547辆清洁能源公交车和出租车。PM2.5年均值比上年削减6.3%，优良空气天数占全年70.2%。推进8个污水处理厂扩容工程建设，在石马河、茅洲河等实施河长制。完成18条内河涌整治。东江水库联网一期工程基本完工。大力创建国家生态城市和国家森林城市，新建改建各类公园33个。

——铁腕整治涉黄涉毒等违法犯罪行为，提升社会治理水平。开展“扫黄”歼灭战，全面清剿涉黄违法行为，坚决铲除保护伞，加强娱乐场所长效管理，有效净化了社会风气。严打涉毒涉众型犯罪，开展“六大专项”及“两抢一盗”打击整治行动，刑事案件破案数上升11.8%。加强劳资纠纷处置，涉及30人以上的劳资突发事件、欠薪逃匿和劳资信访案件分别下降58.7%、73.2%和50%。完善医疗纠纷第三方化解机制，医闹事件下降38%。狠抓安全生产、食品安全、消防安全等专项整治，全年未发生重特大安全事故。

——强化土地监管、财政绩效管理和政府性债务控制。进一步加强依法用地管理，积极配合开展土地审计，着力整改突出问题，规范耕地保护和土地出让管理。完善财政管理制度，加强财政绩效评价，首次公开市本级“三公”支出决算，全面公开300多个行政事业单位“三公”支出预算，试点公开64个市直部门“三公”支出决算。加强政府性债务管控，全面统计核查镇街政府性债务。全市村组资产负债率下降1.8个百分点。

——切实抓好社会民生建设和十件实事。投入252.3亿元发展民生事业，向社会承诺的十件实事圆满完成。开办东莞外国语学校。调增中职学校工科学位4000个。积分制入学公办学位增加11.6%。向民办学校学生发放补助6.7亿元。积极争创全国文明城市“三连冠”。成功入选国家公共文化服务标准化试点。音乐剧《妈妈再爱我一次》获全国“五个一工程”奖，莞产音乐剧影响力不断扩大。入围当当网评比的“非直辖城市图书消费十大城市”，超过13个省成为全国购书记录最多的地级市。亚运会、亚残运会东莞健儿再创夺金新纪录。连续第十年提高基本养老金标准。启动社保跨镇街双定点就医机制。台心医院正式开业。推行家庭医生式服务。发放就业补贴3.2亿元。创业贷款增长13倍。加强流浪乞讨未成年人综合救助保护。向困难群体发放各类补助金10.5亿元。市区内涝整治应急三期工程顺利完成。妥善应对“3·30”大暴雨、登革热等疫情灾害。

——加强对口帮扶与区域协作工作。推进市内“双到”帮扶，欠发达村年纯收入平均增长26%。携手共建深莞惠“3+2”经济圈。深入推进穗莞合作。东莞韶关帮扶合作进入新阶段，“一中心七组团”平台初步形成，引进125个总投资达317亿元项目。东莞揭阳“双到”扶贫工作顺利开展。援疆援藏、对口帮扶广西河池、重庆巫山等工作扎实开展。国防动员、统计审计、人口计生、外事侨务、工青妇幼、民族宗教、档案方志、科普法普、法制、气象、打私等工作有效推进。

——扎实开展群众路线教育实践活动。紧紧围绕“为民务实清廉”主题，针对排查出的31个方面问题和收集的1万多条意见建议，全面落实整改，构建长效机制，着力解决政府系统“四风”问题。聘请第三方明查暗访机关服务质量，试点推进社会评议科长活动。撤销56个市级议事协调机构。清理近九成创建达标活动。加强依法行政。村（社区）法律顾问实现全覆盖。扩大政府购买公共服务范围。开通12345政府服务热线。加快网上办事大厅建设，新推动1599项事项上网办理，努力打通服务群众“最后一公里”。

各位代表！过去一年，全市上下负重前行，克难奋进，经受了严峻考验，迈出了坚实步伐。这些成绩的取得，离不开中央、省委省政府和市委的正确领导，离不开市人大、市政协与各民主党派的监督支持，离不开全市人民、驻莞部队、武警官兵的实干奋进和港澳台同胞、海外侨胞、国际友人的共同努力。在此，我代表市政府，向所有参与、支持和关心东莞经济社会发展的各界人士，表示衷心的感谢和崇高的敬意！

与此同时，我们也清醒看到，东莞资源环境约束趋紧、核心竞争力不强等深层次矛盾还未有效破解，仍然面临不少问题和隐忧：一是部分指标完成不理想。生产总值、消费等与年度增长目标有一定距离，工业投资等增长较慢。二是招商引资势头有所放缓，产能释放尚需时日。新引进重大项目还不够多，已投产项目释放产能的规模效应还不够强。三是公共安全问题仍较突出。各类治安案件及食品安全、生产消防等方面事故时有发生。四是民生热点问题有待加力解决。入学、入户、公交、医疗等公共服务与群众要求还有一定差距。五是政府效能还需进一步提高。一些干部干事创业激情减弱，一些部门审批手续仍较繁琐，提升行政效能还需付出更大努力。针对这些问题，我们将采取积极措施，认真加以解决。

新常态下的新东莞

在新一轮改革发展的关键时刻，习近平总书记提出了中国经济发展进入新常态的重要论断，并深刻阐述了其内涵和特征。新常态是对中国经济新航道、新格局的科学阐释，外在表现为增速放缓与质量提升，实质是经济发展新旧增长动力的转换、新旧发展路径的替代、新旧竞争优势的演化、新旧空间组织的重构以及新旧产业结构的调整过程。这对东莞实现高水平崛起具有重大的现实指导意义。我市作为中国改革开放精彩而生动的缩影，对外开放早、改革起步早、经济发展快，与此相应碰到各种新形势、新情况、新问题也比较早。可以说经济新常态的系列特征在东莞表现得更为深刻明显。具体体现在以下趋势性变化上：

从经济增速看，正逐步从高速增长切换为追求更有质量、效益和后劲的中高速新挡位。这个过程与国际金融危机冲击期、转型升级阵痛期等交叉叠加。近六年来我市有五年“个位数增长”。传统粗放的高速增长已无法再持续，而高效率、低成本、可持续的中速增长成为全市经济发展的新阶段、新目标。

从市场格局看，正逐步从高度外向依赖转为内外两个市场两种资源并重。过去东莞以外资为主、以出口为主、以引进来为主，既成就了开放型经济的特色和优势，也带来了外向依赖过高、经济风险过大的隐患。经过多年努力，全市外向依存度从最高峰的433.8%下降到170.4%，规模以上工业企业内销比重首次超过外销，民营企业纳税额超过六成，外资和民营“比翼齐飞”、出口和内销“两分天下”、引进来和走出去“双轮并驱”的格局已经初步奠定。

从区域协调看，正逐步从镇街小组团向区域大组团一体化发展。随着水乡统筹步伐加快和经济区整合力度加大，随着超3000亿重大项目的平衡布局和市内“双到”扶贫的深入推进，东莞以镇街为单元的小组团格局，正演变为以经济区为主导的大组团格局，行政区划限制被逐步打破，各自为政、重复建设、资源浪费等问题将会有效破解，欠发达镇村的“造血”功能在持续增强，全市一盘棋和区域一体化的协调发展局面正加快形成。

从产业形态看，正逐步从简单的加工贸易向以新技术、新产品、新业态、新模式为特征的“四新”经济方向发展转变。作为东莞经济的发家之本和重要主体，加工贸易在企业形态、技术、品牌等方面的转型升级成效明显，服装、家具等传统优势产业的核心竞争力稳步增强。同时，更多生产要素向“四新”经济集聚，大量新技术广泛应用，智能手机、机器人等新产业初具规模，电子商务、现代物流等新业态、新模式生机蓬勃，国家高新技术企业总数已稳居全省地级市第一位，经济发展正稳步迈入创新驱动的新阶段。

从发展路径看，正逐步从“多轮驱动”向市镇统筹转变。伴随着土地统筹和招商统筹力度的加大，以及利益分配机制的完善，尽管基层仍存在“卖土地、建厂房、收租金”的路径依赖和冲动，但统筹发展的共识不断强化。以往“村村点火”的放养式、多主体、低层次的传统模式，正逐步被“市镇统筹、三级分利”的新模式所替代，镇村集体经济也从以收租为主、存在债务风险的状况，朝着多元投资、增资减债的方向迈出坚实步伐。全市发展层次持续提升。

从改革红利看，正逐步从改革开放的“首创”红利向新一轮全面深化改革的“增创”红利转变。过去30多年，东莞能在没有太多优惠政策的情况下脱颖而出，凭借的是敢为人先精神和招商“一条龙服务”等首创举措。在以往红利衰减式微的新形势下，东莞以自我革命的勇气，又一次在商事制度、项目投资审批等改革中尝到了甜头。全市正掀起新一轮改革攻坚的热潮，不少莞版改革正在逐步增创和释放出新的红利。

从人力资源看，正逐步从“成本洼地”向“人才高地”转变。随着人口红利逐步消减，东莞早期在“孔雀东南飞”背景下形成的劳动力“洼地”效应已全面消退，“求工难”已演变为“招工难”，而且与周边中心城市相比，非但没有人才引进的成本优势，反倒会因为城市配套不完善、人文环境不理想等加重成本。种种新形势倒逼我们必须在政策扶持、工作环境、生活配套等方面下功夫，以待遇引人、以真情待人、以事业留人，努力建设“人才高地”。

从竞争优势看，正逐步从传统的区位和先发优势向激烈竞争下综合新优势转变。随着发展形势的不断变化，我市曾经引以为傲的地理区位等优势已逐步淡化甚至被超越。其他城市数倍于东莞的土地资源优势，北上广深等中心城市和自贸区的“磁吸效应”，制造订单的“去中国化”趋势等，都将是东莞“成长的烦恼”。同时，完善的产业配套成为当前东莞最大的优势，加上良好的经济基础、系统的产业扶持政策，以及近年来着力打造政府服务“加一”和综合成本“减一”的法治化国际化营商环境，东莞正在逐步形成一种新的综合竞争优势。

以上这些趋势性变化，既是东莞发展新常态的外在特征，又是内在动因。新常态为东莞创造了新机遇，也带来了新挑战。如果应对得好，东莞经济将步入平稳健康发展、质量效益提升的新轨道，一幅令人憧憬的新东莞蓝图将在不久的将来成为现实；如处理不好就可能掉入“城市发展陷阱”。我们必须趋利避害，扬长避短，顺势而为，乘势而上，更好地适应新常态、建设新东莞。

适应新常态、建设新东莞，要求我们树立新思维，把握新形势、凝聚新共识。新常态是提质增效的调整，是发展后劲的积蓄。要把思想和行动统一到中央的科学判断上来，深刻认识到东莞发展新常态不可逆转、不容回避。要看清并且承认新趋势、新情况、新问题，不能仍然想着过去铺摊子、卖土地、建厂房的粗放型高速发展模式，那样即使暂时把速度抬上去了也不会持久，相反会使发展中的矛盾和问题进一步积累、激发直至总爆发。要树立和强化新思维，更加注重创新驱动发展，更加注重经济结构战略性调整，更加注重生态建设和富民强市，努力追求实实在在、质量效益提升的经济发展。尤其要通过锐意改革，破除路径依赖，破解发展难题，增创新的发展红利，同时运用法治手段，巩固和深化改革成效，把创新举措转化成工作常态和长效机制，防止体制复归。

适应新常态、建设新东莞，要求我们保持平常心，摒弃“换挡焦虑”、强化“底线意识”。不是经济增速高一点形势就“好得很”，也不是速度下来一点形势就“糟得很”。经济发展速度有升有降是正常的。要认清增速已经换挡的事实，彻底走出高速纠结，摆脱“经济何时才能恢复到危机前速度”等困惑，充分认识到突破资源瓶颈就是构建产业新格局的机遇，战胜复杂经济形势就是赢得城市竞争的机遇，化解深层矛盾就是提升发展水平的机遇，进一步坚定信心，增强发展定力。同时，切实增强忧患意识和责任意识，看到合理经济增速对于促进就业增收、保持投资信心、强化财力保障等的重要意义，采取有力措施确保平稳增长，避免大起大落，努力实现化危为机、逆境突破，推动经济行稳致远。

适应新常态、建设新东莞，要求我们发扬钉钉子精神，坚持主动作为、注重踏实干事。新常态意味着固有发展路径和利益格局的深刻调整，伴随着深层次矛盾和问题的集中爆发，面临着项目引进和用地制约、产能扩张和节能减排、民间投资渠道窄与实体经济融资难等两难困境，一些改革难题亟待攻坚突破，一批重大项目亟需加速建设，招商引资、加工贸易转型、科技金融产业“三融合”等工作的每一步都必须付出巨大努力。必须提振与新常态相适应的干部作风和精神面貌，进一步增强钉钉子的实干精神，破除“乱作为、不作为”现象，抓好决策部署的贯彻落实，抓好目标任务的分解落实，通过一件一件的具体工作、一年一年的务实成效，久久为功，稳步实现长远发展目标，推动东莞在新常态轨道上坚定迈进。

2015年工作安排

2015年是“十二五”的收官之年。展望新的一年，世界部分地区经济增速会略有回升，但总体复苏疲弱态势难有明显改观，经济下行压力仍然很大，外部环境仍然错综复杂，诸多不确定因素将对东莞经济产生较大影响。与此同时，我国经济增长总体处于合理区间，依法治国方针、重大改革举措、“一带一路”战略、新型城镇化等，将创造新的发展红利，激发更多的内需动力，开拓更广阔的外部市场，为我市高水平崛起带来了新机遇。今年政府工作的总体要求是：认真贯彻落实党的十八届三中四中全会、中央经济工作会议、省委十一届四次全会和市委十三届五次全会精神，深入贯彻习近平总书记系列重要讲话精神，以建设“国际制造名城、现代生态都市”为目标，着力加强依法行政，推进重点领域改革，在加快转型升级、强化创新驱动上出实招，在做强制造产业、加速“三重”建设上下功夫，在深化开放型经济体系建设、提升对内对外开放水平上求突破，在创新社会治理、发展民生事业上求实效，注重防范化解风险，更好地适应新常态，努力推动全市经济社会发展行稳致远。

综合考虑各方面因素，今年全市发展的主要预期目标是：生产总值增长8%左右，人均生产总值增长7.5%，市一般公共预算收入增长10%，固定资产投资总额增长12%，社会消费品零售总额增长10%，进出口总额增长5%；先进制造业、高技术制造业增加值占规模以上工业增加值比重分别达47%和37%；研发经费支出占生产总值比重2.3%以上；发明专利授权量增长10%；城市居民人均可支配收入、农村居民人均纯收入分别增长8%和8.5%，居民消费价格涨幅控制在3%左右；全面完成省“十二五”节能减排任务目标。

围绕以上目标要求，重点做好几方面工作：

一、以法治思维引领改革发展，提高依法行政和创新发展能力

党的十八届四中全会作出了全面推进依法治国的重大战略部署。我们必须增强法治思维和创新意识，根据今年市委一号文的要求，将法治贯穿到改革发展的方方面面，使之成为政府工作的指南针和温度计，成为攻坚突破的助推器和保险杠。

深入推进依法行政，提升法治化、国际化营商环境。实行重大行政决策听证和专家咨询论证制度。严格行政处罚自由裁量标准。推行行政机关负责人出庭应诉制度。继续争取地方立法权。加强财政预决算、招投标、“三公”经费等重点领域的信息公开。加快建设企业信用系统。实行重点企业“一对一”挂钩服务，建立企业大数据综合服务平台，搭建“企业问题池”。深入落实系列减负政策，让东莞成为税费收取最透明、综合营商成本最低的城市之一。

深化重点领域改革，争创莞版改革更多亮点。推进外商投资管理服务改革，在所有企业实行“多证联办”。深化商事制度改革，继续在便利化登记和后续监管两端发力。落实项目投资改革举措，推广直接落地改革试点经验。稳妥发展混合所有制经济，全面深化国企新一轮改革。在公共基础设施项目投资领域，加快实行政府与社会资本合作（PPP）模式。推动全国农村综合改革示范点建设，完善集体资产交易和监管机制，深化股权制度改革。推进城乡土地生态利用制度综合改革，开展不动产统一登记和土地承包经营权确权。探索实施镇街排污总量指标有偿调配制度。

大力推进行政权力清单化、行政审批标准化。编制部门权责清单，实现审批事项一单尽收，赋予唯一代码，凡不在清单上的审批事项，一律不得保留。率先在项目投资领域推行行政审批标准化，逐步覆盖所有审批领域，核减并明确每个审批事项所需资料、流程和办理时限。推动能上网审批事项尽可能上网，实行全程大数据管理，着力提高网上办理率和办结率。

加强政府自身建设，以问题为导向推动作风持续改善。巩固群众路线教育实践活动成果，将改进会风文风、杜绝铺张浪费等经验做法制度化。针对居住证办理、户籍变更等常办事项，开展专项督查，解决擅自增设环节、中介变相收费等问题。积极开展第三方评估，扩大暗访和作风评议范围，深入整治“庸懒散拖”，倒逼机关作风改善。严格执行八项规定，加强政府系统廉政建设，打造为民务实清廉的法治政府。

二、实施东莞制造2025战略，建设中国制造样板城市

当前，全球制造业正在迈入数字化、网络化、智能化的新时代。我们必须坚定制造业强市方向，按照今年市政府一号文的要求，大力实施东莞制造2025战略，推进智能制造、服务型制造、创新制造、优质制造、集群制造、绿色制造“六大工程”，努力将东莞建设成为中国制造样板城市。

掀起制造业智能化改造热潮。加快实施“机器换人”计划，选择一批重点项目示范带动智能化改造。在模具、服装等传统行业推动数控一代机械产品运用。引导企业加快实施电机能效提升和注塑机节能伺服技术改造。预留用地指标，优先解决投资强度每亩500万元以上的技改项目，对拆除重建、加层等符合条件的技改项目，新增建筑面积不再征收土地出让金。

推动制造业“扩链强链”。依托智能手机省市共建基地，推动全产业链集聚发展。依托机器人产业基地和“机器换人”庞大市场，引进培育机器人龙头企业，加快形成集群优势。依托省数控一代示范镇建设，整合科研机构、装备制造企业和应用单位资源，培育和完善数控产品产业链。依托服装、家具、五金模具等专业镇，用好专业市场、品牌展会、行业创新平台等资源，推动传统优势产业占据上下游高附加值链。

打造制造业的“东莞质量”和“东莞标准”。推进质量强市建设，推动检测和认证业创新发展，将东莞建成华南地区重要检测基地。鼓励企业参与标准制定，规划重点行业标准路线图，抢占智能机械手、新能源汽车、智能手机等行业制高点。开展工业产品质量专项整治，加强质量监督抽查。加快工业设计等生产性服务业发展，提高“东莞制造”的品牌含金量。

进一步加大对金融业发展的支持力度，推动民营资本与先进制造业深度融合。整合金融资源，通过境内外上市、股权融资等，扩大企业直接融资比例。引导民间资本参与或设立产业基金、创投基金，投资制造业项目和技术。探索建立新型民营股权投资基金。建立先进制造业项目大数据资源库，定期举办对接活动，推动资本与项目在碰撞融合中迸发出制造业发展新活力。大力推行设备融资租赁，为急需设备更新的中小企业提供融资服务支持。

三、大力发展新技术、新产品、新业态、新模式，增强创新驱动发展能力

加强新型研发机构和科技企业孵化器建设。加快大学创新城二、三期工程建设。与北京航空航天大学等国内外知名院校加强合作，进一步推动新型研发机构进驻并发挥作用。加快散裂中子源项目建设，发展关联配套产业。实施孵化器“双提升”与“全覆盖”行动，加快香市科技园等大型孵化器建设。促进科技创新服务业发展。鼓励开展各类股权众筹与创客路演活动。

加快发展电子商务、配送物流和港口经济。引导更多专业镇建立电商平台，鼓励企业用好第三方电商推动线上线下融合发展。鼓励企业收集用户需求，生产个性化产品，打造互联网工厂。全面推行电商企业集群注册。完善跨境电商公共服务平台功能，构筑跨境电商专业园区体系。推进全国城市共同配送试点。以虎门港为载体，打造保税加工、现代物流、临港产业三位一体的综合性开放平台。

促进城市商务商贸功能进一步完善。全面启动南城国际商务区北区开发，引进一批企业总部和高端服务业。结合轨道交通TOD开发，探索以空中连廊等方式促进商贸资源整合，推动中央商圈和各镇成熟商圈功能强化、布局优化。探索建立新型商业购销平台和物流集散中心。完善厚街作为全市会展核心镇的功能，提升会展产业集中度。

加快旅游业发展和酒店业转型升级。突出“项目强市、旅游富民”，出台旅游业发展规划，深入挖掘莞香旅游资源，重点打造虎门销烟、明清古村落、岭南水乡等精品线路，力争引进若干个旅游大项目，打响东莞古迹游、水乡游、会展游、工业游等“十大旅游”品牌。通过“异业同盟”等手段加快酒店业转型升级，提升东莞酒店业品牌形象。

四、构筑对内对外开放新格局，提高开放型经济核心竞争力

建设海上丝绸之路先行市。依托 “一带一路”战略，继续办好海博会，积极拓展与东盟、非洲、南美等地区的经贸文化合作，探索与沿线国家共建产业园区，推动企业加快走出去步伐。加快台湾高科技园、两岸生物产业基地、中以产业园、中英低碳产业园等建设。主动对接融入广东自贸区。

加快转变外经贸发展方式。推进国家加工贸易转型升级试点城市建设，深入实施加工贸易增效计划。探索建设国际贸易“单一窗口”。以区域通关一体化为契机，推动物流跨区域整合和功能延伸。大力发展服务贸易和服务外包。强化加博会、漫博会、台博会、国际科技合作周等品牌展会对产业转型升级的带动作用。

多层次、全方位、多渠道主动出击招商。建立招商引资“项目源”大数据。围绕机器人、智能手机、电子商务等产业，瞄准重点地区登门招商。充分发挥莞商和在莞商会作用，加强以商引商。鼓励和帮扶现有企业增资扩产。在美国硅谷建立商务代表处，探索设立国际合作引导基金，力争将东莞打造为“硅谷先进制造中心”。

集聚境内外优秀人才，营造进得来、留得下、用得好的最佳人才环境。进一步放宽人才入户限制，创新人才引进机制，探索实行企业自评人才入户。研究引进高端退休“银色人才”。加强技能人才培养，推行中职教育校企深度融合的现代学徒制，加强重点企业的订单式人才培养和引进。完善城市生活配套和公共服务供给，让人才更愿意留在东莞。

五、按照“建设一批、投产一批、储备一批”的要求，让重大项目成为经济结构战略性调整的强力引擎

落实“五个一”、明白卡、销号制等制度，加速重大产业项目落地开花结果。建立重大项目建设“问题池”，加大市镇领导挂钩督导力度。对征地拆迁难、管线迁改慢等共性问题进行集中会审，对个性问题及时协调解决，对推而不动、无法开工的坚决取消重大项目资格、收回土地指标。重点推动中集物流等总投资605.8亿元的项目动工，加快华为终端总部、步步高研发生产基地等建设，力争年内投产重大项目13个，新增产值346.4亿元。

全力加强土地等资源要素保障。力争统筹连片土地3万亩，盘活存量土地、处置闲置土地1.3万亩，形成“熟地”目录，强化招商用地保障。鼓励金融机构加大对重点项目的信贷投放力度。加快完善项目水电气等基础设施及周边生活配套，协助解决员工子女读书等问题，全方位优化投产项目的跟踪服务。

完善重大项目引进和管理机制，形成项目储备梯队。健全绩效考核与奖励机制，掀起重大项目引进新热潮。全市一盘棋优化重大项目布局，建立重大项目统筹流转和利益共享机制，实行镇街（园区）共建“飞地招商”税收分成。推广凤岗利用分散厂区承接重大项目的做法。对未完全满足既定条件、但对我市产业发展具有战略性意义的重大项目，实行“一事一议”认定。

六、以国家新型城镇化综合试点为契机，打造现代生态都市

强化“产城融合”理念，以重大平台引领“一中心四组团”格局完善。依托市区、松山湖、生态园打造中心组团，完善功能配套，强化城市中心区辐射带动。加快松山湖大道沿线开发。依托水乡打造西北组团，启动水乡新城建设，推进疏港大道延长线等路网工程。依托虎门、长安和长安新区打造西南组团，完善虎门港码头设施和航线，争取长安新区上升为省级战略，对接湾区经济。依托塘厦、清溪、凤岗等打造东南组团，建设现代产业发展集聚区。依托“一区一镇”打造东北组团，加快银瓶创新区建设和招商，强化常平镇辐射带动，建设产城融合发展示范区。

做好步入城市地铁时代的各项准备工作。加快轨道交通建设，全面建成2号线，开通运营莞惠城际轨道常平至惠州段，启动有轨电车试验段建设。完善地铁站点周边及东莞火车站、虎门白沙站、东莞东站等交通枢纽功能配套，统筹开发地上地下空间，建设一批高开发强度、多功能复合的城市综合体。加快城市更新改造，优先安排成片改造用地指标，抓好5个示范片区项目，鼓励“工改工”和新型工业楼宇建设。严格查处违法用地和违建行为。

加快电力设施、信息网络等基础建设。打好电网规划建设大会战，力争投产110千伏及以上输变电工程19项，进一步强化电力保障。超前建设光纤、基站等通信设施，实现重点公共区域WiFi全覆盖。启动深圳外环高速东莞段、环莞快速路三期等工程，加快虎门二桥等项目建设，推动石大公路大修等项目建成通车。圆满完成省下达我市的高速公路建设任务。

打造山清水秀的宜居城乡。积极创建国家生态市、森林城市、水生态文明城市。推进美丽幸福村居建设。加强“小山小湖”保护利用，建成不少于100个社区公园。发展现代农业，加快高标准基本农田建设。推进植物园二期工程。完善森林公园配套。推进节能减排财政政策综合示范城市建设。推广河长制、涌长制。加快挂影洲中心涌水环境综合整治。加快污水处理厂改扩建及次支管网工程，力争石马河、茅洲河水质进一步改善。深入开展大气污染防治行动。把环保限行区域扩大到全市，淘汰全部黄标车。加快水乡“两高一低”企业整治和退出。加大环境监察执法力度。加强东江及联网水库水源保护，实施安全饮用水工程。

有序加快农业转移人口市民化进程。加快制定人口发展规划，依法确定合理的人口迁入条件和规模。优化积分制入户政策。增加10%积分入学学位供给。建立与居住年限等挂钩的基本公共服务提供机制，探索制定“同城共享”批次清单，让居住证持有人逐步享有与户籍人口同等的基本公共服务。完善政府向社会组织购买服务机制，为市民提供更好公共服务。

七、积极创新社会治理，营造安定和谐、文明有序的发展环境

严打各类违法犯罪，时刻绷紧“安全弦”。保持对涉黄涉赌行为的高压严打态势，强化娱乐场所长效管理。严打涉毒涉黑、“两抢一盗”等犯罪。每个镇街完成1—2个治安重点区域或行业整治。加强刑事侦查队伍能力建设。加快公安指挥中心及情报平台、第二看守所第三监区配套工程建设。发挥视频监控在治安管理中的作用。深化出租屋及流动人口管理服务。严格落实安全生产工作责任制，强化隐患排查整改，加强应急救援演练，开展消防安全、食品安全、极端天气安全等专项整治，切实维护群众生命财产安全。推进和谐劳动关系示范区创建工程，力争企业参与率达70%。健全工会律师团工作机制。完善劳资纠纷预警与应急处置机制，严厉打击欠薪逃匿犯罪行为。加强医患纠纷第三方调解。

推进交通大整治和“微治理”。开展为期一年的交通大整治，抓好镇际村际公路等建设，完善路政管养，明晰行车指引，清拆标牌违建，做好绿化美化，改善过镇过村公路脏乱差堵现象。开展交通拥堵路段“微治理”，通过增划转弯车道、增设中间护栏、查处占道通行、优化路口交通组织等精细化手段，为市民提供安全顺畅的通行环境。

开展社区网格化管理试点，创新基层社会治理。整合村级政务服务中心、综合服务中心和综治信访维稳工作站等，打造统一的综合服务管理平台。选取若干镇村作为试点，划分网格作为基本治理单元，整合综治维稳、城市管理、公共安全、市场监管、自治管理等队伍，在网格内实行“一组多能、一员多职”。加强网格内信息采集和上传，推动网格管理员和各职能部门共享一个系统、一套数据，切实提高基层管理效能。

八、带着对群众的真挚感情，切实办好民生社会事业

着力解决医疗卫生、公共交通、垃圾处理等群众关注的热点问题。加快市人民医院分院、市儿童医院等建设。在公立医院实行医药分开，所有药品零差价销售。优化公立医院绩效考核。提高社区卫生服务水平。整合跨镇公交资源，优化完善公交线网，提高群众公交满意度。加强出租车承包管理，严肃查处违规经营行为。推进“数字城管”建设。加快麻涌、横沥等环保热电厂改造建设，完善生活垃圾处理生态补偿机制。进一步加强内涝整治。推进家禽集中屠宰、生鲜上市。

办好十件实事，带动民生社会事业全面发展。创建省推进教育现代化先进市。提高公办中学经费标准，对民办学校教师发放从教津贴。推进民办学校标准化建设。构建慕课创新教育平台，选取若干学校开展翻转课堂、在线教育等新型教学试点。完善异地中考政策，取消民办学校小升初考试。推进社保扩面征缴，争取让全市社会养老保险退休人员悉数进入全省统筹。低保标准提高至每人每月610元。为户籍老年人购买意外伤害险。非莞籍老人纳入免费乘公交范围。推动残疾人事业发展。完善12345热线功能。促进就业创业。加大市内扶贫力度。加强对口帮扶韶关、揭阳和援疆援藏工作。加快深莞惠“3+2”经济圈建设。进一步深化穗莞合作。积极争创全国双拥模范城“八连冠”。

以贴近群众为宗旨，促进文化文艺事业百花齐放。实施文化惠民工程，创建国家公共文化服务标准化试点城市。大力弘扬优秀传统文化，挖掘重大历史题材，推进电视剧《袁崇焕》拍摄。完善基层文化设施配套。设立专项资金扶持民办博物馆发展。深化全国文明城市创建工作，培育践行社会主义核心价值观，强化未成年人思想道德教育和法治教育。

以高水平承办苏迪曼杯为契机“办赛事、办城市”。努力办好我市承办的首个重要国际体育赛事。全力做好赛事接待、环境布置和绿化美化工作，展现整洁有序、清新亮丽的城市形象。以赛事宣传为切入点，进一步创新城市营销，运用更加符合网络新时代特点的各种渠道和手段，加强城市整体包装推广，全面改善和提升东莞城市形象。

各位代表，2015年是“十二五”规划的收官之年，也是“十三五”发展的谋划之年。“十三五”是我国实现第一个百年目标、全面建成小康社会的关键时期，也是东莞加快转型升级、实现高水平崛起的决胜时期，编制好“十三五”规划具有重大而深远的意义。各级各部门必须强化全局意识，树立战略思维，准确把握东莞发展的阶段特征和时代要求，科学编制全市“十三五”规划纲要，以及科技、交通、教育等18项重点专项规划和各镇街、园区的“十三五”规划，确保各项规划的前瞻性、科学性和可操作性，注重与城乡总体规划、土地利用总体规划等规划的融合，注重各级各类规划的无缝衔接，精心编制好全市未来五年发展的总纲领、路线图和时间表。

各位代表！我们正处在变革与奋进的时代。唯有积极投身新一轮改革开放的伟大实践，才能让东莞再立潮头、再谱新篇。让我们紧密地团结在以习近平同志为总书记的党中央周围，在中共东莞市委的坚强领导下，团结和带领全市人民，主动适应新常态，奋力建设新东莞，在高水平崛起的征程上阔步向前！
